# The Relevance and Resilience of Evo‐Devo in 2025: The Biennial Meeting of the Pan American Society for Evolutionary Developmental Biology

**DOI:** 10.1002/jez.b.70020

**Published:** 2026-03-22

**Authors:** Mark Rebeiz, Jennifer A. Brisson, Chris Amemiya, D. Nathaniel Clarke, Tetsuya Nakamura, Leslie Pick, Patricia Schneider, Yuichiro Suzuki, Christina Zakas, Karen D. Crow

**Affiliations:** ^1^ Department of Biological Sciences University of Pittsburgh Pittsburgh Pennsylvania USA; ^2^ Department of Biology University of Rochester Rochester New York USA; ^3^ Department of Molecular and Cell Biology University of California Merced Merced California USA; ^4^ Department of Biology University of Miami Coral Gables Florida USA; ^5^ Department of Genetics Rutgers University Piscataway New Jersey USA; ^6^ Department of Entomology University of Maryland College Park Maryland USA; ^7^ Department of Biological Sciences Louisiana State University Baton Rouge Louisiana USA; ^8^ Department of Biological Sciences Wellesley College Wellesley Massachusetts USA; ^9^ Department of Biological Sciences North Carolina State University Raleigh North Carolina USA; ^10^ Department of Biology San Francisco State University San Francisco California USA

The 2025 biennial meeting of the Pan American Society for Evolutionary Developmental Biology (PASEDB), held July 22–25 at the University of Miami in Coral Gables, Florida, was a dynamic gathering of 112 researchers dedicated to studying how developmental processes shape evolutionary change across the tree of life. PASEDB was founded to promote the study of evolutionary developmental biology (evo‐devo) and to provide an inclusive forum for collaboration (Abouheif and Sears 2015), communication, and education across the Americas and beyond. Since its inaugural meeting in 2015 at UC Berkeley (Edgar and Chinga 2015; Lesoway 2016), PASEDB has become a cornerstone of the American evo‐devo community.

Biennial meetings continue PASEDB's tradition of engaging with current challenges, both scientific and existential, while strengthening connections across its membership and advocating for the future of evo‐devo. The 2025 meeting took place amid a rapidly shifting political climate and an increasingly uncertain scientific funding environment in the United States. Despite these challenges, the PASEDB Council elected to move forward with the meeting, reaffirming the Society's commitment to fostering collaboration, maintaining momentum in the field, and providing a forum for scientific engagement during a period of uncertainty. As a result of these factors, overall attendance, especially from countries outside of the United States, was lower than at previous meetings. To partially address this, plenary speakers from Latin America were invited to give talks via zoom to the group meeting in person in Miami. The diminished international presence underscored the importance of sustained efforts to promote scientific exchange across borders, a core value of the evo‐devo community.

## 1 Promoting Evo‐Devo Research

The plenary sessions of the meeting were held with all attendees present in one room, allowing all attendees to collectively enjoy the talks and topics spanning the breadth of the discipline. The scientific program highlighted the conceptual, empirical, and methodological diversity of evo‐devo. Presentations spanned core concepts such as the evolution of developmental mechanisms, the origins of novelty, and phenotypic plasticity, while showcasing an array of methodological approaches. Equally notable was the diversity of study systems represented, with work on emerging and non‐traditional models such as placozoans and upside‐down jellyfish presented alongside more established systems. Together, these contributions highlight the power of evo‐devo to generate insights by combining conceptual breadth, technical innovation, and biological diversity. Below we highlight some of the overarching themes that emerged across these talks.

A particularly strong theme centered on discovering the developmental genetic basis of intra‐ or interspecific variation. Talks by Heather Hines (PI, Penn State) and Christina Zakas (PI, North Carolina State University) provided compelling examples. Hines presented work on the genetic basis of a red‐black pattern transition in three co‐mimetic bumble bee species in the western United States, demonstrating that the closely related species each acquired their color forms independently, but did so by targeting the same cis‐regulatory locus in an abdominal Hox gene (Hines et al. 2025). The work of Zakas focused on the marine polychaete *Streblospio benedicti*, which produces two heritable larval morphs that differ dramatically in egg size, embryogenesis, and larval morphology. By identifying morph‐specific gene expression differences, particularly within the chitin‐synthesis pathway underlying chaetal development, this work pinpointed candidate genes contributing to life‐history and morphological differentiation within this species. Together, these studies showed how evo‐devo integrates genotype, development, and phenotype to explain ecologically relevant variation.

Another prominent theme that emerged from the main session was the incorporation of environmental influences into evo‐devo frameworks, emphasizing how developmental systems interpret external cues to produce phenotypic outcomes. This perspective was seen in a talk by Erik Ragsdale (PI, Indiana University), which focused on the molecular architecture of developmental plasticity of feeding structures in the nematode *Pristionchus pacificus*. Using replicated experimental evolution, Ragsdale and colleagues examined how resource competition drives shifts in the induction of the predatory mouth morph, which allows starved nematodes to consume competitors. Their results revealed genetic parallelism across independently evolved populations, including repeated emergence of a specific cis‐regulatory variant affecting a transcription factor (Levis and Ragsdale 2025).

A significant developmental response to the environment is the ability of an organism to regenerate after injury. Talks throughout the conference showed the power of evo‐devo approaches to investigate the differences between species which can regenerate and those that can't, as well as the many ways that regeneration manifests in different organisms. James Nowotny (PhD student, University of Maryland) shared his work on whole‐body regeneration in annelids, which revealed somatic de‐differentiation and proliferation at nerve endings. This contrasts with the migrating pluripotent stem cells implicated in flatworms. Gabriela Lima (PhD student, Louisiana State University) presented a transcriptomic and ATAC‐seq comparative analysis of highly regenerating vertebrates (axolotl, African lungfish, and Senegal bichir), identifying regulatory elements commonly associated with regeneration in these disparate lineages.

One of the most frequent evolutionary transitions is trait loss. Federico Brown (PI, Universidade de São Paulo) reported on the reduction of eyes in cave planarians, providing evidence for a mechanism of organ size reduction via decreased specification of eye‐cell progenitors during homeostasis and regeneration (Saad et al. 2025). In tetrapods, digit loss is common, and the most frequently lost digit is digit 1 (the thumb). Jacob Scott (PhD student, University of Florida) leveraged an expansive ‐omics dataset to identify regulatory elements associated with recurrent digit 1 loss.

In cnidarians, the medusozoa life stage is an ancestral trait that has been lost in multiple lineages, such as hydrozoa. Paulyn Cartwright (PI, University of Kansas) presented work that implicated Hox gene expansions in the origins of the medusozoan form and associated the loss of the Hox gene Tlx with the loss of the free‐swimming medusa stage in hydra.

As evo‐devo benefits from its wide spectrum of study systems, our science becomes more amenable to new bioinformatic approaches as they become available. Athula Wikramanayake (PI, University of Miami) presented work on the *Nematostella* Wnt pathway, notably using AlphaFold to predict and identify β‐catenin binding motifs in the *Nematostella* Axin homolog, suggesting a promiscuous origin to this important signaling pathway protein interaction (Sun et al. 2024). Rodrigo Nunes‐da‐Fonseca (PI, Federal University of Rio de Janeiro) presented a comparative analysis of the small ORFome of holometabolous and hemimetabolous insects by comparing bioinformatic predictions coupled with RNA‐seq data. This revealed both evolutionarily static and variable parts of the small ORFome (Guerra‐Almeida et al. 2025; Webster et al. 2026a).

The nervous system is one of the final frontiers of biology, and the meeting highlighted how evo‐devo has played an important role in understanding the links between evolution of the nervous system and the diverse behaviors that exist within and between species. Néva Meyer on behalf of Johnny Davila‐Sandoval (PhD student, Clark University) presented work on how the central nervous system of the spiralian annelid *Capitella teleta* is formed (Webster et al. 2026b). Combining classical blastomere isolations with transcriptomics, they identified genes from a lineage‐specific homeobox class as differentially expressed across the embryonic axis and that the canonical Wnt signaling pathway is important for neural induction in the CNS. Vikram Chandra (Postdoc, Harvard University) presented a quantitative study of the brain and behavior of an early‐branching acoel worm, showing that this brain uniquely lacks internal anatomical and functional regionalization. This work suggests that the first brains may have had similar organization, with regionalization evolving secondarily (Chandra et al. 2025).

On a different note, Jasmin Camacho (Postdoc, Stowers Institute) presented a talk that showed us the biomedically‐translational potential of evo‐devo studies. In studying the evolution of exaggerated traits, Jasmin chose to investigate metabolic resistance in nectar feeding bats. Combining lab and field research, she found that these bats continuously maintain a high level of blood glucose, a level that would be considered dangerous in humans (Camacho et al. 2024). She proposed that rewiring of insulin‐responsive signaling pathways, particularly in skeletal muscle, underlies their ability to tolerate extreme hyperglycemia without metabolic disease.

One of the greatest benefits of scientists from different parts of a field to gather is to exchange progress on the latest methodologies. Single cell RNA‐seq appeared in many talks, highlighting the broad adoption of this approach to distill complex developing tissues into clusters of cell types based on gene expression differences. We began to see talks with spatial transcriptomics, showing the promise of localizing every gene's expression pattern spatially within the same tissue. Genomic approaches continue to gain momentum including ATAC‐seq to monitor open chromatin, and high throughput genotyping to map individual variants in genetic crosses. And colorimetric whole mount *in situ* hybridization approaches are currently being replaced with much more high‐resolution fluorescent approaches such as hybridization chain reaction (HCR). Because evo‐devo is a hybrid amalgamation of several interrelated fields, we are constantly updating our toolkit of approaches, drawing from the constituent fields that make up evo‐devo.

A notable feature of the scientific program was the inclusion of four, day‐long taxon‐specific satellite symposia on the opening day. The symposia provided forums for deeper field‐specific scientific discussions and reflections on resource needs within specific communities. The Arthropod Satellite highlighted taxonomic and conceptual diversity, with talks spanning butterfly wing color patterning, tardigrade limb development, beetle horn morphogenesis, and aphid wing dimorphism, while emphasizing themes of developmental bias, regulatory evolution, and systems drift. A lively conversation underscored priorities around resource sharing, data and protocol accessibility, and ambitious comparative research. The Fish Symposium brought together researchers working across lineages to explore the developmental and genetic bases of novel traits, integrating comparative anatomy, functional genomics, and advanced imaging to address innovations ranging from feeding structures and appendages to craniofacial traits and tooth evolution. The Spiralian Satellite showcased how emerging model systems are rapidly gaining powerful molecular and functional tools, enabling fresh insights into axis formation, RNA localization, and lineage‐specific novelties. A dedicated discussion session focused on community‐building efforts centered on shared genomic resources, collaborative annotation, and trainee engagement. Finally, the Non‐Bilaterian Satellite meeting highlighted how new molecular and single‐cell approaches are transforming early‐branching animal lineages into key systems for understanding the origins of cell types, developmental programs, and life cycles prior to the rise of bilaterians. Together, these satellite symposia set an energetic and intellectually rich tone for the remainder of the meeting.

## 2 Recognizing Excellence in our Field at all Career Stages

As a society, one of our major goals is to encourage early‐stage researchers, while also recognizing the contributions of longstanding members of our community. We do this at our meetings through judged poster and talk awards, a postdoc symposium, and through dedicated award sessions. The PASEDB Postdoctoral Excellence Award was presented to Kevin Deem of the University of Rochester for his work on gene regulatory elements in pea aphids. The young Investigator Award was conferred to Igor Scheider (Louisiana State U) who presented his work on fin regeneration in fishes. The Rudy Raff Pioneer Award was conferred to Günter Wagner (Yale University), who presented on the future of the field from the perspectives of his long career of work in many diverse systems. He emphasized that evo‐devo (or his preferred “devo‐evo”) has a bright future ahead and has a special place to contribute to evolutionary biology, particularly with the advent of single cell technologies and research into comparative cell type evolution. Chris Amemiya (UC Merced) received the PASEDB Service award for his career‐long dedication to the field (as a past NSF program officer and past president of the society). His talk provided heartwarming encouragement to perpetuate “ohana” in our community ‐ the practice of caring and sharing and spreading the aloha spirit. Awards for talks and posters were presented to student and postdoctoral presenters.

## 3 Promoting the Exchange of Ideas

Beyond the formal scientific program, a central mission of PASEDB is to foster networking, discussion, and a sense of community among its members. The Coral Gables meeting offered multiple opportunities to do so. Two well‐attended poster sessions provided a forum for researchers at all career stages to share ongoing work in an informal and interactive setting. A community‐building workshop created space for broader conversations about the relevance and value of evo‐devo, with discussion emphasizing the importance of educational outreach, particularly efforts aimed at inspiring and engaging younger generations. Participants also reflected on ways scientists can more effectively communicate the importance of science education and research support to local and federal decision‐makers, underscoring the role of the evo‐devo community as advocates as well as researchers. These exchanges were complemented by social events that bookended the meeting, with receptions on the first and last evenings encouraging relaxed conversation and connection; the opening reception concluded with a fun karaoke session, while the final evening featured a poetry slam. Together, these events reinforced PASEDB's commitment to cultivating an engaged and collaborative evo‐devo community.

## 4 Concluding Thoughts

In summary, PASEDB's 2025 Biennial Meeting was a memorable and successful gathering that brought together investigators across career stages, taxa, and subdisciplines. Evo‐devo is a relatively small niche, with most researchers working in relative isolation within their home institutions. For many, this meeting is the singular forum where one's research is fully appreciated and reinforced by like‐minded scientists united by a common goal. For those who yearn for this kind of connection, we hope you join us at the 2027 Biennial Meeting to be held June 15–18th at Cornell University in Ithaca, New York. We anticipate continued opportunities to reconnect with colleagues, exchange ideas, and further advance the field of evo‐devo, while warmly welcoming new members to join and help grow the PASEDB community.

## Conflicts of Interest

The authors declare no conflicts of interest.

## 6.2 Peer Review

For transparency, the peer review documents associated with this article are available at https://doi.org/10.1002/jez.b.70020.

## Data Availability

This article is a commentary and does not include original data. Therefore, no data are available.
